# Extracellular vesicles secreted by highly metastatic clonal variants of osteosarcoma preferentially localize to the lungs and induce metastatic behaviour in poorly metastatic clones

**DOI:** 10.18632/oncotarget.9781

**Published:** 2016-06-02

**Authors:** Rebecca Macklin, Haolu Wang, Dorothy Loo, Sally Martin, Andrew Cumming, Na Cai, Rebecca Lane, Natalia Saenz Ponce, Eleni Topkas, Kerry Inder, Nicholas A Saunders, Liliana Endo-Munoz

**Affiliations:** ^1^ The University of Queensland Diamantina Institute, The University of Queensland, Translational Research Institute, Brisbane, Australia; ^2^ Therapeutics Research Centre, School of Medicine, University of Queensland, Brisbane, Australia; ^3^ Queensland Brain Institute, The University of Queensland, Brisbane, Australia; ^4^ Australian Institute for Bioengineering and Nanotechnology, The University of Queensland, Brisbane, Australia

**Keywords:** osteosarcoma, metastasis, extracellular vesicles, interclonal communication, pre-metastatic niche

## Abstract

Osteosarcoma (OS) is the most common pediatric bone tumor and is associated with the emergence of pulmonary metastasis. Unfortunately, the mechanistic basis for metastasis remains unclear. Tumor-derived extracellular vesicles (EVs) have been shown to play critical roles in cell-to-cell communication and metastatic progression in other cancers, but their role in OS has not been explored. We show that EVs secreted by cells derived from a highly metastatic clonal variant of the KHOS cell line can be internalized by a poorly metastatic clonal variant of the same cell line and induce a migratory and invasive phenotype. This horizontal phenotypic transfer is unidirectional and provides evidence that metastatic potential may arise via interclonal co-operation. Proteomic analysis of the EVs secreted by highly metastatic OS clonal variants results in the identification of a number of proteins and G-protein coupled receptor signaling events as potential drivers of OS metastasis and novel therapeutic targets. Finally, multiphoton microscopy with fluorescence lifetime imaging *in vivo*, demonstrated a preferential seeding of lung tissue by EVs derived from highly metastatic OS clonal variants. Thus, we show that EVs derived from highly metastatic clonal variants of OS may drive metastatic behaviour *via* interclonal co-operation and preferential colonization of the lungs.

## INTRODUCTION

Osteosarcoma (OS) is the most common malignant primary bone tumor in children and is characterized by a high degree of vascularization, genomic complexity, inter- and intra-tumoral heterogeneity, chemoresistance and early pulmonary metastasis [1–4]. Treatment with aggressive, multi-agent neo-adjuvant chemotherapy and limb-salvage surgery has increased the survival of patients with localized disease, but for the majority of patients who progress to pulmonary metastasis, current treatment strategies have been ineffective [5, 6]. To improve outcomes for metastatic OS patients we need to understand and target mechanisms involved in driving metastatic progression.

The process of tumor growth, progression and metastasis relies on a complex network of inter-cellular, intra-cellular and distant cell signaling events and interactions [7]. These include signaling between cancer cells and stromal cells in the tumor microenvironment, cancer cell-cell communication, and interaction with distant organs targeted for metastatic spread. Although numerous pathways have been shown to participate in these processes it is now clear that extracellular vesicles (EVs) secreted by cancer cells may be key mediators of cell-to-cell communication and cancer progression [8, 9].

Extracellular vesicles, including exosomes, microvesicles and apoptotic bodies, are membrane-bound nanoparticles, 30–1000 nm in size, which are secreted by all cell types, including cancer cells [10, 11]. EVs display cell membrane markers from the secreting cell and carry a defined array of proteins, lipids, mRNA and miRNAs that differ according to their cell of origin [12]. EVs from various cell types induce genotypic and phenotypic changes in recipient cells and change the outcome in a number of diseases, including cancer, *via* the transfer of their protein and nucleic acid cargo [8]. For example, EVs secreted by fibroblasts in the tumor microenvironment can stimulate the motility and progression of breast cancer cells [13]. On the other hand, tumor-derived EVs can also transfer their cargo to cells in the tumor microenvironment, a process which has been shown to induce angiogenesis, to modulate anti-tumor immune responses and to prepare the pre-metastatic niche [14–17]. For example, in lung tissue, EVs secreted by melanoma cells initiate the degradation of the extracellular matrix and promote vascular leakiness [10]. Importantly, when the transfer of EVs occurs between cancer cells, it induces signaling events in the recipient cells that promote growth and survival [10].

To date, the literature reporting the role of EVs in sarcomas is limited. One study has reported the isolation and characterization of EVs from Ewing's sarcoma cells [18], and another study with 143B osteosarcoma cells has revealed that they secrete EVs capable of stimulating osteoclastogenesis in the surrounding bone microenvironment [19]. However, it is unknown whether OS-derived EVs can participate in intra-tumoral communication, specifically, in inter-clonal cooperation which could modify the metastatic behaviour of OS. In addition, extracellular vesicles have been proposed as significant and useful clinical prognostic biomarkers due to their presence in the biological fluids of cancer patients, and their increasing levels with advancing disease [20, 21]. Yet, no literature exists examining their prognostic value in osteosarcoma.

In this study, we show that the transfer of vesicles from highly metastatic OS subclones induces a change in the poorly metastatic subclones to a more migratory and invasive phenotype. This increase in metastatic properties is unidirectional. In addition, we profile the exosomal proteome and identify a suite of known metastasis effectors. Finally, we show that vesicles derived from highly metastatic OS subclones localize specifically and preferentially to the lungs.

## RESULTS

### Clonal variants differing in metastatic potential exist within an established OS cell line

**Figure 1:**
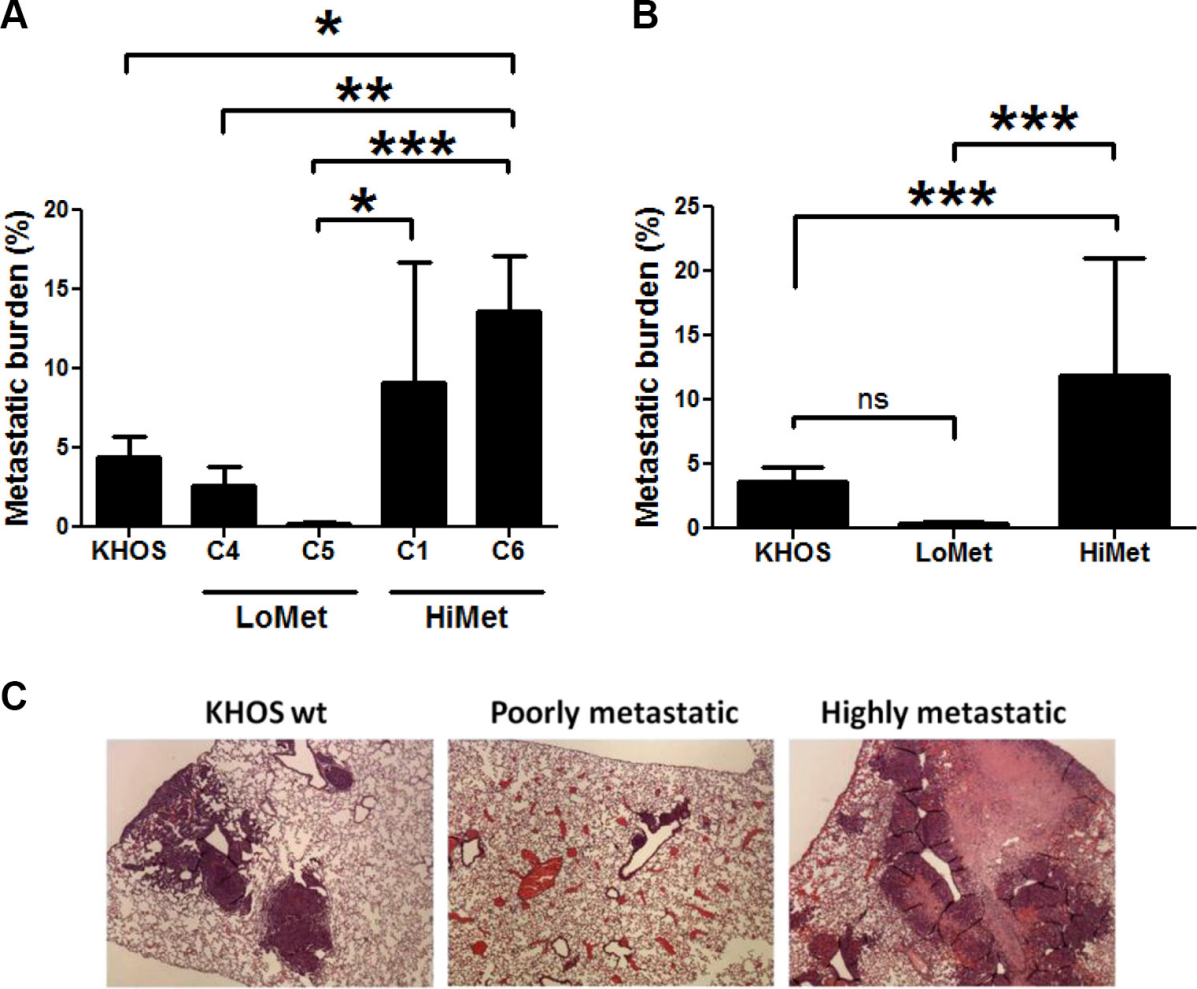
OS tumor heterogeneity Mice were injected intrafemorally with 5 × 10^4^ cells in a 10 μL volume of PBS and tumours allowed to grow to 10 mm diameter before being sacrificed. (**A**) Metastatic burden in mice injected orthotopically with the metastatic KHOS parental cell line and single cell clonal variants C1, C4, C5 and C6, derived from KHOS. Data presented as mean +/− SD from 2 experiments. *n* = 10. Statistical analysis: One-way ANOVA (F = 7.14, *P* = 0.0002), and Tukey's Multiple Comparison Test for *P* < 0.05. (**B**) Average metastatic burden of experiment shown in (A). One-way ANOVA (F = 22.77, *P* < 0.0001), and Tukey's Multiple Comparison Test for *P* < 0.05. (**C**) Representative H&E stained lung sections of mice at the time of sacrifice following injection with KHOS (wt), highly metastatic HiMet-C6 or poorly metastatic LoMet-C4 cells, showing OS lung lesions. Magnification: ×10. Experiment was performed twice with 5 mice/group each time.

We have previously described the isolation of clonal variants from an established OS cell line (KHOS). Using our published mouse model of OS metastasis [22], we selected six clonal variants (termed C1–C6) at random and individually injected them intra-femorally into 6-week-old BALB/c nude mice, as described. All clonal variants gave rise to tumors in the injected animals within 4–9 weeks post-injection. All clonal variants were used at a very early passage after expansion and used in two independent mouse experiments performed 3 months apart. In both experiments the behaviour of the clones was reproducible in terms of tumor formation and metastasis. The six clonal variants separated into highly metastatic (HiMet-C1 and C6) or poorly metastatic (LoMet-C2, C3, C4 and C5) when compared to the parental cell line (Figure 1A and 1B; C2 and C3 not shown). The metastatic foci formed by different clonal variants also displayed different characteristics, with poorly metastatic clonal variants giving rise to small and distinct metastatic foci, and highly metastatic variants giving rise to large, often necrotic foci which spread over the lung tissue and merged with each other to form large areas of metastasis (Figure 1C). These data show that variants exist within an established tumor-derived metastatic OS cell line and that they differ in their metastatic activity.

### Extracellular vesicles secreted by HiMet OS modify the phenotype of LoMet OS

**Figure 2:**
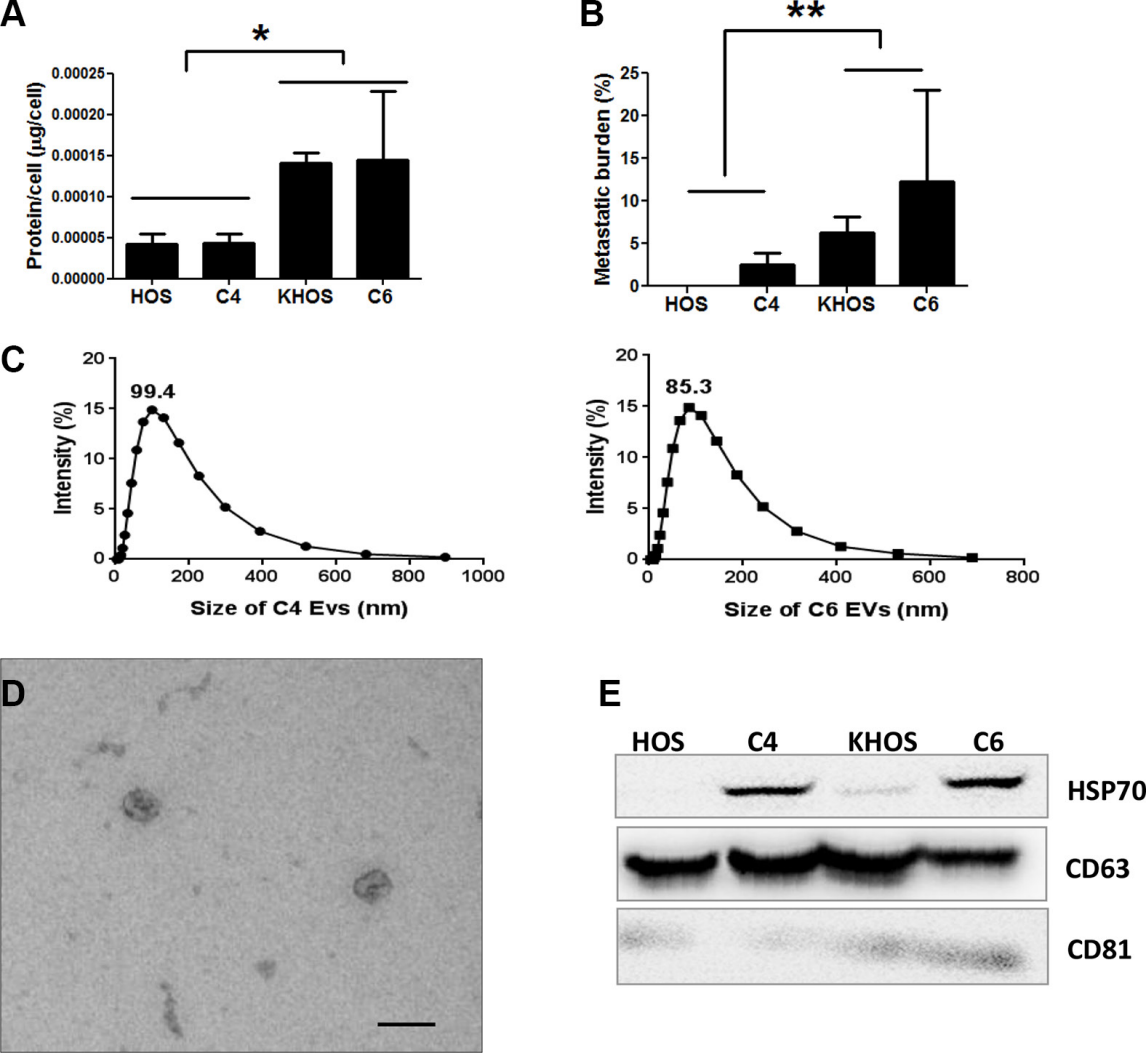
Characterization of OS extracellular vesicles EVs were isolated from conditioned medium collected from 24 h cultures of various OS cell lines or clones grown in the presence of FCS-EV-free medium. (**A**) Quantity of EVs in unfiltered medium, estimated as EV protein secreted by metastatic KHOS cells, the clonal variants HiMet-C6 and LoMet-C4, and non-metastatic HOS cells. Protein levels normalized to the number of cells in culture. Data presented as mean +/− SD of triplicate determinations from 3 experiments. Statistical analysis: 1-way ANOVA (F = 6.40, *P* = 0.0039), and Tukey's Multiple Comparison Test for *P* < 0.05. (**B**) The metastatic potential of the cells in (A) was determined by orthotopic injection into mice. Lungs were collected when primary tumors reached 10 mm, FFPE and sectioned. Metastasis was quantified as % area of lung covered with metastatic lesions/total area of lung for each mice, Data presented as mean +/− SD of 2 experiments, *n* = 10. Statistical analysis: 1-way ANOVA (F = 5.99, *P* = 0.0015), and Tukey's Multiple Correction Test for *P*< 0.05. (**C**) Measurement of the size distribution by % intensity of a filtered and diluted preparation of EVs containing 40 μg/mL of LoMet-C4 (left panel) or HiMet-C6 (right panel) EVs in a Zetasizer 3000HSa. Data presented as mean of two preparations. (**D**) Electron microscopy of a filtered preparation of EVs derived from HiMet-C6 (< 200 nm), showing the classic doughnut shape reported for exosomes. Bar: 100 nm. (**E**) EV protein isolated from the OS cell lines (KHOS, HOS) and the clonal variants (HiMet-C6, LoMet-C4) was electrophoresed and immunoblotted against classic exosome markers, CD63, CD81 and HSP70. A representative blot from 3 experiments is shown.

To investigate whether OS cells were capable of secreting EVs and to examine whether the levels of EV secretion in OS correlated with metastatic potential, we isolated extracellular vesicles from a 24 h culture of a metastatic cell line, KHOS, and a non-metastatic cell line, HOS, as well as from the clonal variants HiMet-C6 and LoMet-C4, grown in FBS EV-free medium. EVs can be isolated by serial ultracentrifugation or commercial precipitation techniques. Both techniques have been used successfully but can also co-purify contaminants that can impact biological activity [12, 23, 24]. Therefore, we compared the ability of HiMet-C6 EVs isolated using a commercial kit or serial ultracentrifugation to increase the migration of LoMet-C4 cells (Figure S1A) or parental KHOS cells (Figure S1B). Both preparations of EVs increased migration of target cells similarly. This is consistent with published studies showing that commercial reagents or ultracentrifugation protocols produced EVs of equal purity and functional quality [14, 25–27]. Moreover, in a proteomic analysis of soluble factors secreted by metastatic and non-metastatic OS cells, we failed to detect VEGF, PDGF or TGFβ [28], thus excluding these as possible soluble contaminants in our EV preparation that could be contributing to the increase in metastasis. Thus, for the rest of our study we used a commercial precipitation kit (ExoQuick) for EV enrichment. Initial studies showed that EVs could be isolated from non-metastatic HOS cells, poorly metastatic LoMet-C4 clones, highly metastatic HiMet-C6 clones, and the metastatic parental cell line KHOS (Figure 2A and 2B). HOS and LoMet-C4 clones contained low levels of protein/donor cell whereas the metastatic C6 clone and KHOS cell line contained 3-fold more protein/donor cell (Figure 2A). Particle size distribution of the HiMet-C6 and LoMet-C4 vesicles revealed very little difference in size between the two preparations, with peak sizes of 99.4 nm and 85.3 nm for LoMet and HiMet, respectively (Figure 2C). However, due to the limitations of Zetasizer particle size analysis for polydisperse EV preparations, we sought confirmation of the size of our vesicles by fixing the HiMet-C6 EV preparations in paraformaldehyde for electron microscopy. The images revealed numerous vesicles 80–90 nm in diameter displaying a typical doughnut shape which has been previously reported for some exosomes (Figure 2D) [29, 30]. Finally, we performed immunoblotting of EVs < 200 nm isolated from HiMet-C6, LoMet-C4, the non-metastatic cell line, HOS, and the metastatic cell line, KHOS, against a panel of classic exosome antibody markers: HSP70, CD63 and CD81. All preparations were positive for CD63, the most commonly identified (82%, http://www.exocarta.org) [31] marker in exosomes, as well as for CD81 and HSP70. The expression of the latter was much higher in the extracellular vesicles isolated from the clonal variants C6 and C4, than in those isolated from the cell lines (Figure 2E). These data demonstrate that OS cells secrete abundant extracellular vesicles and that the level of EV secretion correlates with the metastatic potential of the cell. Specifically, highly metastatic OS cells secrete 3-fold more vesicles than poorly metastatic cells.

Extracellular vesicles secreted from tumor cells into their microenvironment are known to induce phenotypic changes in stromal target cells [32]. Evidence is also beginning to accumulate showing that this phenotypic transfer can occur between cancer cells [33, 34]. However, there is limited evidence showing that EVs secreted by HiMet subpopulations within a heterogeneous tumor can transfer a metastatic phenotype to neighboring LoMet subpopulations (i.e. interclonal cooperation), and no evidence in osteosarcoma.

**Figure 3:**
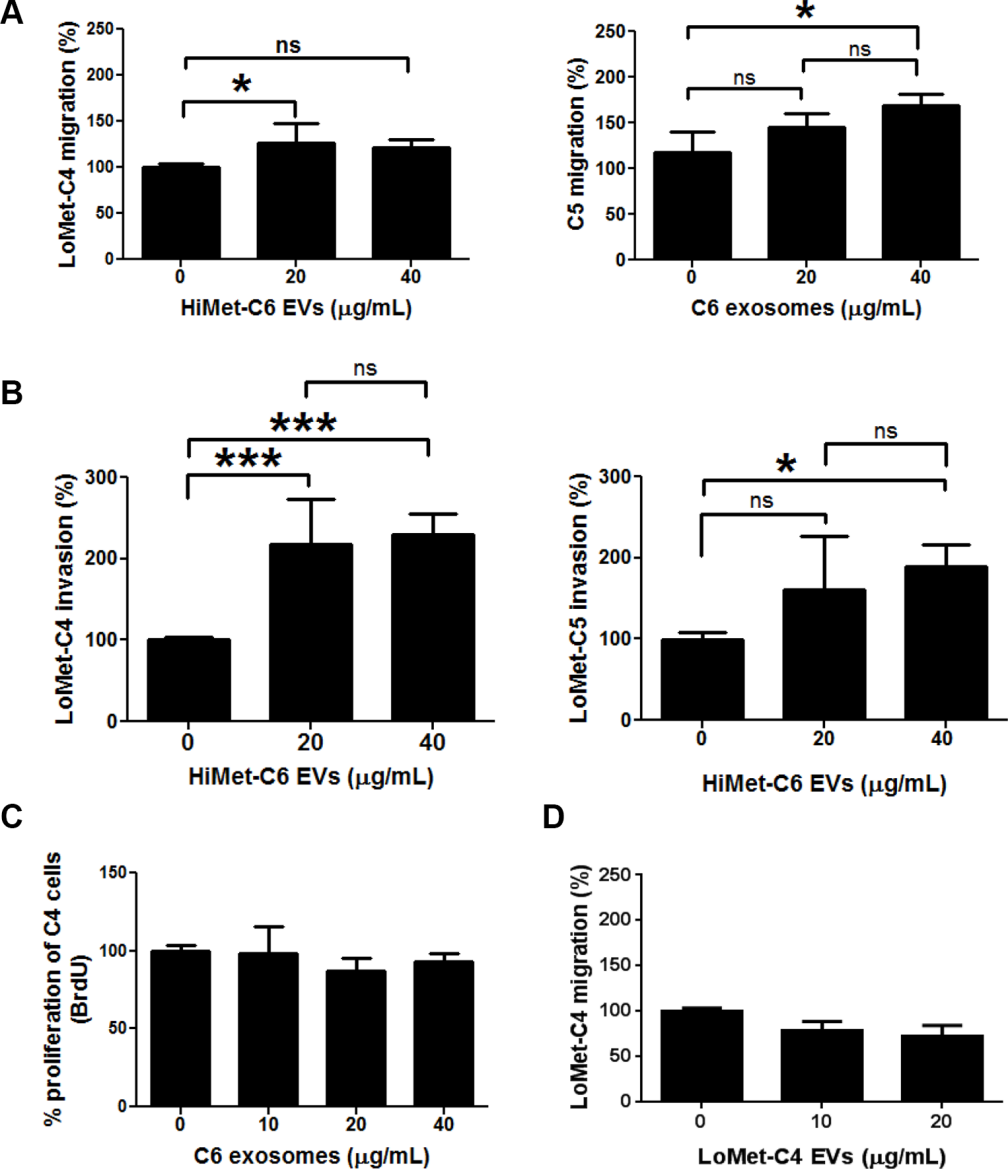
Extracellular vesicles secreted from HiMet cells increase *in vitro* migration and invasion of LoMet cells EVs were prepared from HiMet-C6 or LoMet-C4 clones and incubated with LoMet-C4 or C5 clones for 24 hr. (**A**) Increase in the migration of LoMet-C4 and C5 in the presence of EVs secreted by a HiMet-C6. *1-way ANOVA (F = 3.97–4.13, *P* = 0.032–0.04), Tukey's MCT for *P* < 0.05. (**B**) Increase in the invasion of LoMet-C4 and C5 in the presence of EVs secreted by HiMet-C6. *1-way ANOVA (F = 4.15, *P* = 0.036), Tukey's MCT for *P* < 0.05. (**C**) Increase in migration of LoMet-C4 cells in the presence of HiMet-C6 EVs is not due to the proliferation of LoMet-C4 cells, measured as DNA synthesis (BrdU incorporation). 1-way ANOVA with Tukey's MCT = ns. (**D**) LoMet-C4-secreted EVs do not contribute to the increase in migration of LoMet-C4 cells. 1-way ANOVA with Tukey's MCT = ns. Data presented as mean +/− SD from triplicate determinations of at least 2 experiments. Bars: SD.

**Figure 4:**
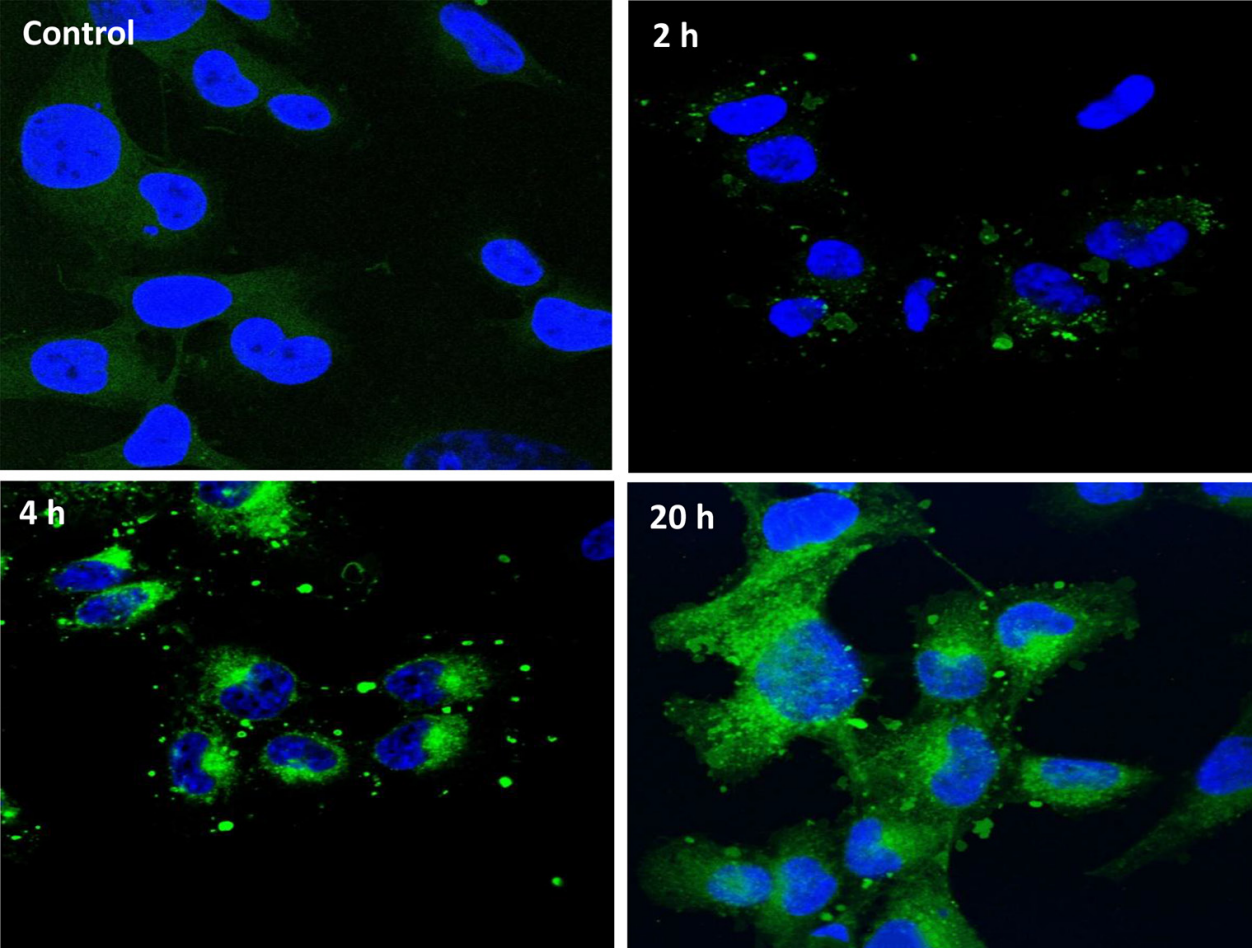
HiMet-C6 EVs are internalized by LoMet-C4 cells EV membranes from HiMet-C6 cells were labelled with PKH67 dye as described in “Materials & Methods” and 20 μg/mL of labelled EVs (green) incubated with LoMet-C4 cells (nuclei stained blue with DAPI) for the indicated times. Images are representative of 3 independent experiments. Visualization was performed by confocal microscopy at 63× magnification (Zeiss LSM 510 Meta).

We investigated whether HiMet-C6 EVs could change the migratory and invasive potential of LoMet-C4 cells. We performed *in vitro* migration and invasion assays using EVs at concentrations of 20 and 40 μg/mL. HiMet-C6 EVs significantly increased the migration (*P* = 0.032–0.04) and invasion (*P* = 0.036) of LoMet-C4 and Lo-Met-C5 cells (Figure 3A and 3B). HiMet EVs from another clonal variant, C1, also significantly (*P* < 0.0001) increased the invasion of LoMet-C5 (Figure S2A). We confirmed that the increases in migration and invasion were not due to an increase in LoMet-C4 proliferation in the presence of HiMet-C6 EVs during the assay (Figure 3C). In addition, we also confirmed that EVs secreted by the LoMet-C4 cells did not contribute to the increase in migration of the LoMet-C4 cells in a paracrine fashion (Figure 3D). In contrast, LoMet-C4 and C5 EVs did not increase migration and invasion of HiMet-C1 or C6 (Figure S2B–S2D), indicating that the ability to transfer a migratory and invasive phenotype is unidirectional and unique to EVs secreted by highly metastatic subpopulations. To understand this process more fully we considered the possibility that LoMet OS cells could take up EVs secreted by HiMet OS cells. We labelled HiMet-C6 EVs with the membrane dye, PKH67, prior to incubation with LoMet-C4 cells. Our data shows that HiMet-C6 EVs begin to be internalized by LoMet-C4 cells after 2 h, and that the number of internalized vesicles increases as time progresses, persisting after 20 h (Figure 4). Combined, these data provide evidence for the transfer of migratory and invasive phenotypes from highly metastatic to poorly metastatic osteosarcoma subclones through the release and uptake of EVs.

### The HiMet EV protein cargo plays a role in metastasis

We performed proteomic analysis of EVs secreted by HiMet-C6 and LoMet-C4. Extracellular vesicles secreted by cells grown in the absence of serum have been shown to differ in protein composition from those secreted by cells grown in the presence of serum [35]. For this reason, and to mimic the conditions under which the phenotype changes had been observed in functional migration and invasion assays, we chose to grow cells in EV-free medium containing 10% FCS.

**Figure 5:**
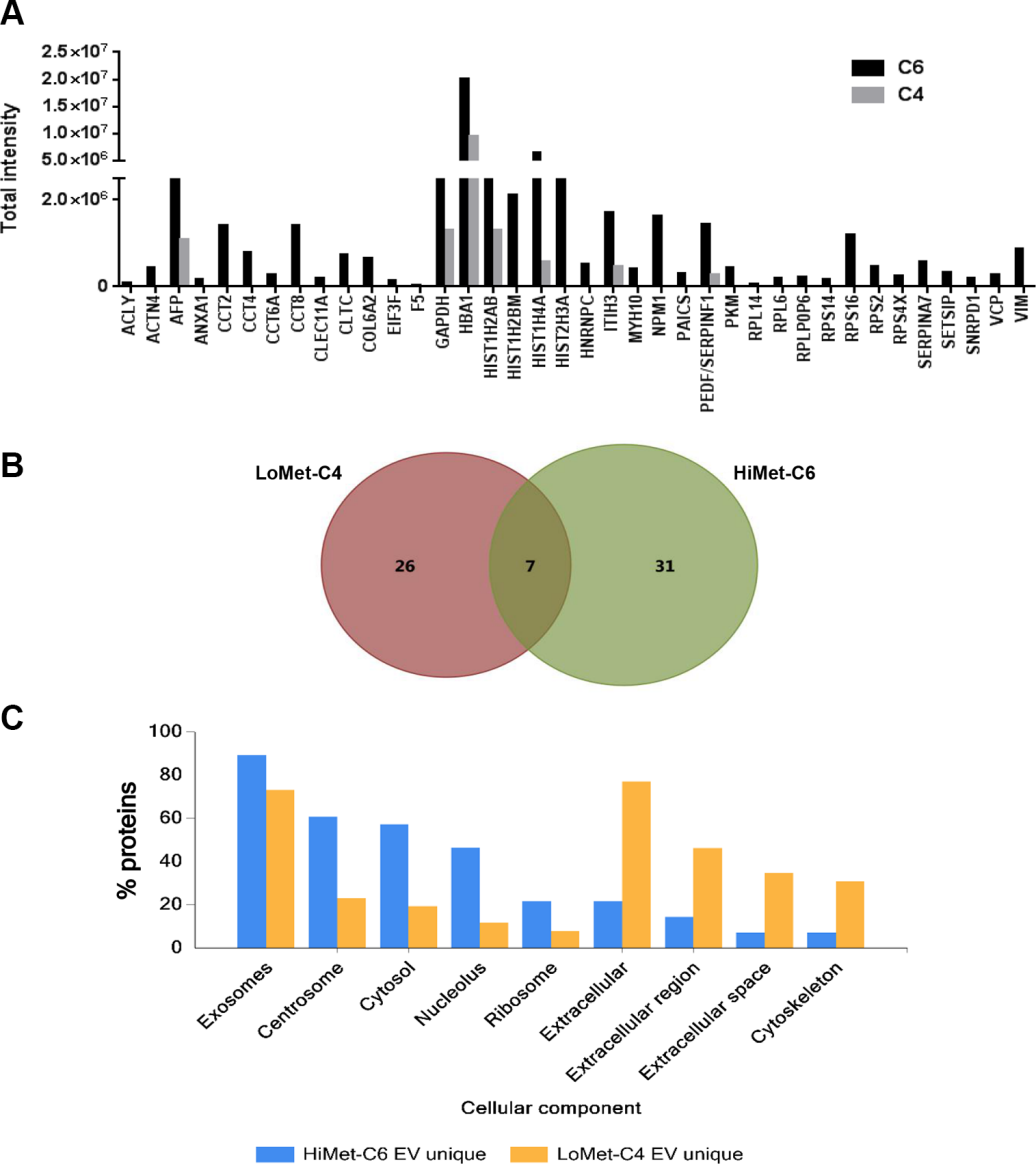
Protein cargo of HiMet-C6 and LoMet-C4 EVs EV preps from FCS-EV-free medium derived from 3 biological replicates of LoMet-C4 and HiMet-C6 EVs were prepared and subjected to MS as described in “Materials & Methods”. Total peak intensity was calculated from the extracted ion chromatogram of each peptide precursor. All peptides have a false discovery rate < 0.5%. The graph (**A**) shows 38 peptides exclusively present in HiMet-C6 EVs or present with a fold change ≥ 2.0 in HiMet-C6 EVs compared with LoMet-C4 EVs. (**B**) Venn diagram showing 26 proteins exclusively expressed in LoMet-C4 EVs, 31 proteins exclusively expressed in HiMet-C6 EVs, and 7 proteins in common. (**C**) Comparison of the cellular component of the proteins found uniquely in HiMet-C6 and LoMet-C4 EVs.

**Figure 6:**
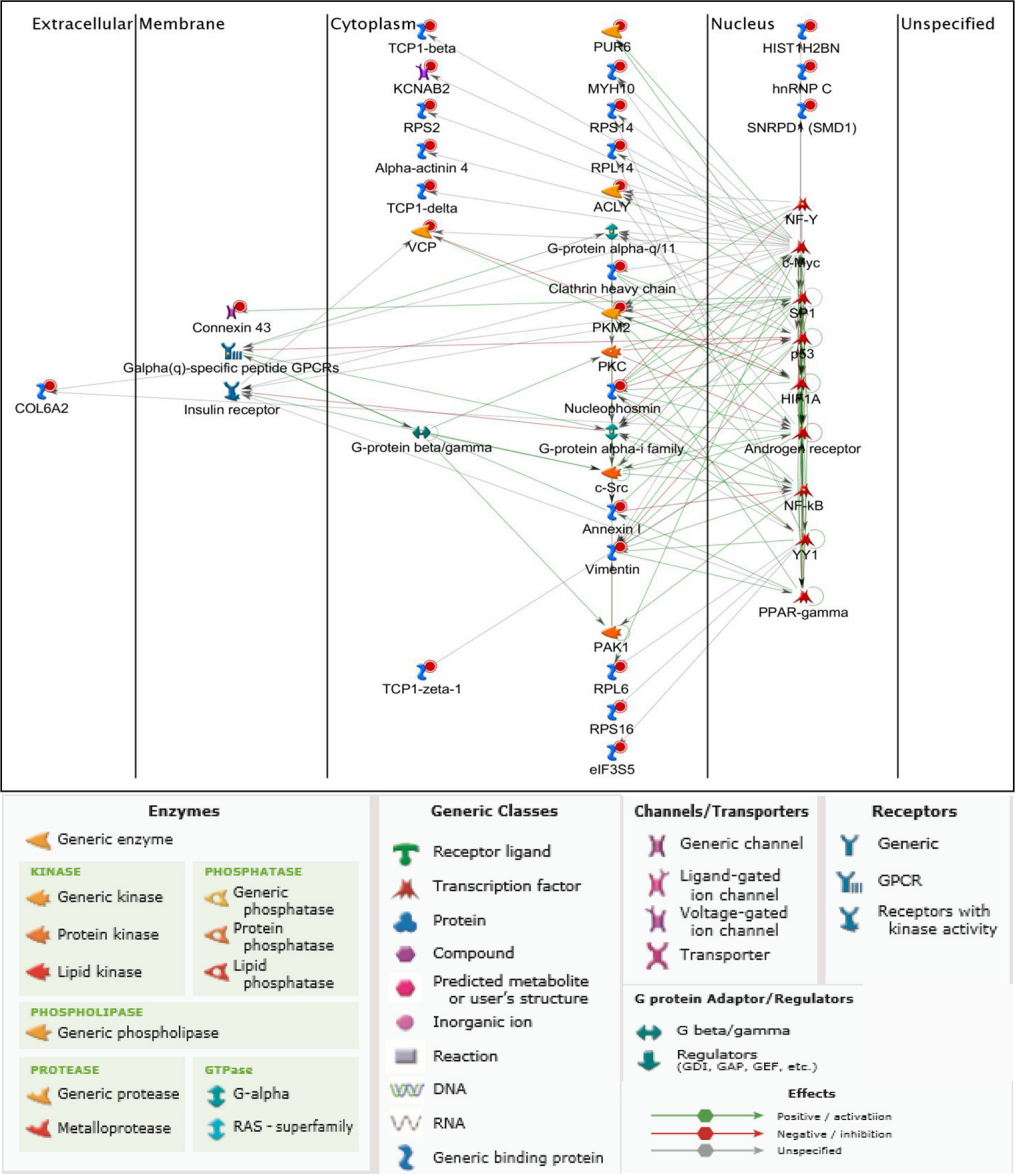
G-protein coupled receptor signaling by HiMet-C6 EV protein cargo Based on the proteomic profile of the HiMet-C6 clones we generated a network map using receptor target modelling in Metacore GeneGo. Red circles indicate proteins uniquely present in HiMet-C6 vesicles.

We performed false discovery rate (FDR) validation after mass spectrometry analysis to remove a number of peptides belonging to background bovine serum proteins. After FDR validation (FDR < 0.5%), the analysis identified 64 proteins in HiMet-C6 EVs (Table S1) and 37 proteins in LoMet-C4 EVs (Table S2). These proteins were present in at least two of the three biological replicate MS runs. Of the 64 proteins found in HiMet-C6 EVs, 31 were unique to these vesicles and 7 were present at levels 2-fold greater than in LoMet-C4 vesicles (Figure 5A and 5B). Proteins shared by HiMet and LoMet vesicles included alpha-fetoprotein (AFP), glyceraldehyde-3-phosphate dehydrogenase (GAPDH), hemoglobin subunit alpha (HBA1), histones H2A and H4 (HIST1H2AB, HIST1H4A), inter-alpha-trypsin inhibitor heavy chain H3 (ITIH3) and pigment epithelium-derived factor (PEDF). FunRich (Functional Enrichment Analysis Tool) [36] was used to compare the cellular component of the proteins found uniquely in HiMet-C6 and LoMet-C4 EVs. Whilst HiMet-C6 EV proteins were mainly found in exosomes, centrosomes, cytosol and nucleolus, LoMet-C4 EV proteins were mainly of extracellular and cytoskeletal origin (Figure 5C). This served to further highlight the differences between vesicles secreted by the two subclones. The most abundant proteins present in HiMet-C6 which were present at a fold change ≥ 2 compared to LoMet-C4 were HIST1H4A (FC = 11.14), PEDF (FC = 5.28), ITIH3 (FC = 3.64) and HIST1H2AB (FC = 3.55). Of the 31 most abundant proteins which were present in HiMet-C6 EVs but not in LoMet-C4 EVs, 16 (51.6%) have been shown to be involved in metastatic progression in various cancers including OS (Table 1). These include nucleophosmin (NPM1), several chaperonin complex proteins (CCT2, 4, 6A and 8), vimentin (VIM), clathrin heavy chain (CLTC), collagen alpha-2(VI) chain (COL6A2), heterogeneous nuclear ribonucleoproteins C1/C2 (HNRNPC), pyruvate kinase (PKM), alpha-actinin 4 (ACTN4), myosin-10 (MYH10), multifunctional protein ADE2 (PAICS), valosin containing protein (VCP), annexin A1 (ANXA1) and ATP-citrate synthase (ACLY). Receptor target modelling (MetaCore data-mining and pathway analysis, Thomson Reuters) of the 31 unique HiMet-C6 proteins in the list revealed that 21/31 of the proteins (67.7%) are involved in G-protein coupled receptor signaling events (GO Processes) (Figure 6) known to play critical roles in tumor growth and metastasis [37]. More recently, a link between GPCR signaling, extracellular vesicles and metastasis has been published [38].

**Table 1:** HiMet-C6 extracellular vesicle proteins associated with metastatic progression

Protein	Role in metastasis	References
NPM1	Associated with a variety of signaling pathways regulating cell proliferation and apoptosis. Involved in tumorigenesis as suppressor and oncogene. Overexpressed in a number of cancers. Associated with colorectal cancer migration and invasion.	[54, 75–77]
CCT2 CCT4 CCT6A CCT8	Possible role in cell proliferation and tumorigenesis (ER-positive breast cancer, gallbladder carcinoma and colorectal cancer).	[78–81]
VIM	Involved in attachment, migration, and cell signaling. Expression correlates with increased metastatic disease, reduced patient survival and poor prognosis across multiple tumor types.	[55–58]
CLTC	Increasing evidence for the role of trafficking pathways in metastasis by directing cell motility.	[82–84]
COL6A2	Associated with poor survival in ovarian cancer.	[85]
HNRNPC	Controls aggressiveness of glioblastoma cells through the regulation of PDCD4.	[86]
PKM	Overexpressed in a number of cancers. Associated with aggressive clinicopathological features and poor prognosis in HCC.	[86–91]
ACTN4	Encoded by metastasis-related gene. Protein expression is closely associated with the invasive phenotypes of cancers.	[92–97]
MYH10	Direct targeting by miR-200a inhibits cell migration and tumor growth in meningiomas and lung adenocarcinomas.	[98, 99]
PAICS	Encoded by anti-apoptotic oncogene. Prognostic biomarker for aggressive lung adenocarcinoma. Increased expression associated with disease progression and poor prognosis. Altering expression modulates cell proliferation and invasion.	[100, 101]
VCP	Inhibition suppresses OS metastasis.	[66–68]
ANXA1	Deregulation associated with development, invasion, metastasis, occurrence and drug resistance of cancers. Might specifically function as a tumor suppressor or a tumor promoter candidate for certain cancers depending on the particular type of tumor.	[102]
ACLY	Expression is associated with advanced stage and prognosis in gastric adenocarcinoma.	[103]

### Extracellular vesicles secreted by HiMet OS subpopulations increase chemotaxis and invasion of LoMet subpopulations

**Figure 7:**
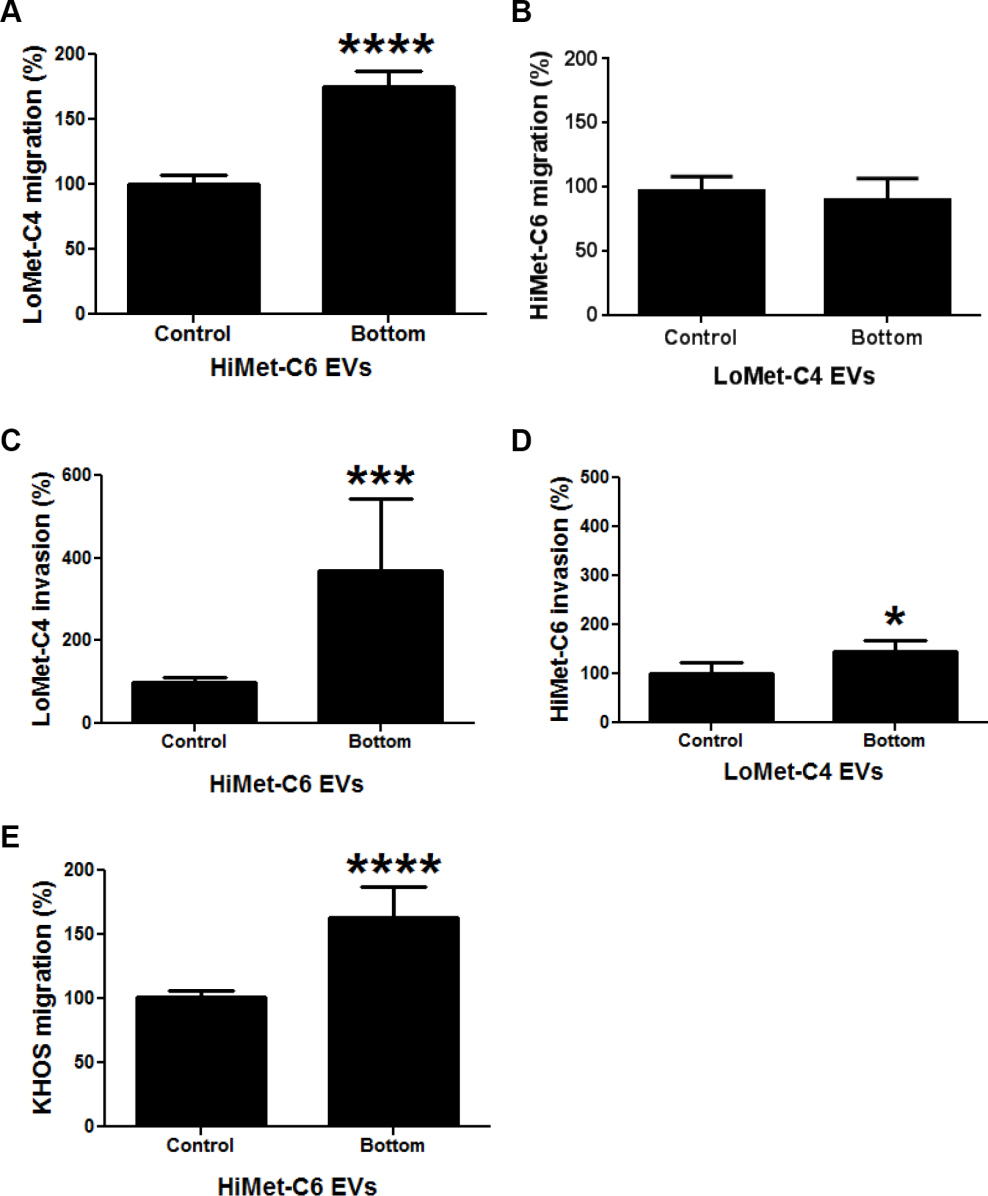
Chemotactic properties of HiMet EVs EVs were isolated from FCS-EV-free medium of HiMet-C6 or LoMet-C4 clones and 20 μg/mL EVs placed in the bottom chamber of transwell chambers. (**A**) Increase in the migration of LoMet-C4 in response to HiMet-C6 EV chemotaxis. ****P < 0.0001. (**B**) LoMet-C4 EVs do not induce an increase in the migration of HiMet-C6 cells by chemotaxis. (**C**) Increase in the invasion of LoMet-C4 in response to HiMet-C6 EV chemotaxis. ***P = 0.0003. (**D**) LoMet-C4 EVs induce a modest response in invasion of HiMet-C6 cells by chemotaxis. *P = 0.015. (**E**) Increase in the migration of the heterogeneous, metastatic parental cell line, KHOS, in response to HiMet-C6 EV chemotaxis. ****P < 0.0001. Data presented as mean +/− SD of triplicate determinations from at least 2 experiments. Two-tailed *P* value from an unpaired *t* test.

Extracellular vesicles have been shown to release chemokines that increase the invasion of target cells, an event which may contribute to the formation of a pre-metastatic niche [39–41]. To test whether HiMet OS EVs could increase the chemotactic properties of LoMet OS cells, we used the same *in vitro* surrogate assays of metastasis described above. However, this time EVs were placed in the bottom chamber of the transwell, and migration or invasion was allowed to proceed for 24 or 48 h, respectively. HiMet-C6 EVs significantly increased the chemotactic migration (*P* < 0.0001) and invasion (*P* = 0.0003) of LoMet-C4 cells (Figure 7A and 7C). In contrast, LoMet-C4 EVs had no effect on the chemotactic migration of HiMet-C6 cells (Figure 7B) and only modestly increased (*P* = 0.015) their chemotactic invasion (Figure 7D). In addition, HiMet-C6 EVs significantly (*P* < 0.0001) increased the chemotactic migration of the heterogeneous parental cell line, KHOS (Figure 7E). Similar results were obtained with HiMet-C1 and LoMet-C5 cells (Figure S3). Thus, our data show that extracellular vesicles secreted by highly metastatic subpopulations can increase the chemotactic migration and invasion of poorly metastatic OS cells.

### HiMet-C6 extracellular vesicles preferentially colonize lung tissue *in vivo*

**Figure 8:**
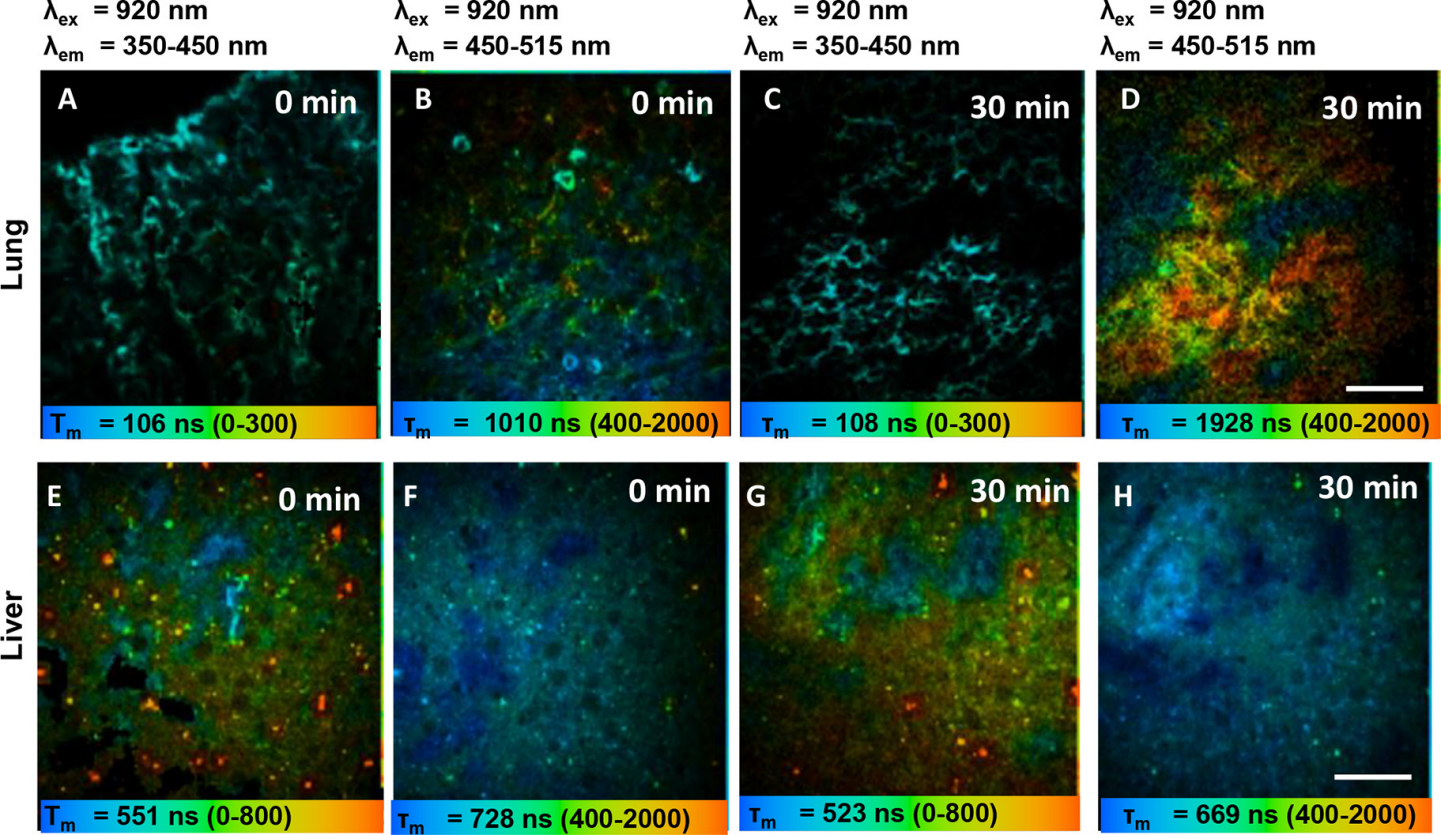
HiMet-C6 EVs migrate to the lung but not the liver (**A**–**D**) Pseudocolored τm fluorescence lifetime image of PKH67-labelled HiMet-C6 EVs in mouse lung before (A, B) and 30 min after bolus injection (C, D). (**E**–**H**) Pseudocolored τm fluorescence lifetime image of PKH67-labelled EVs in mouse liver before (E, F) and 30 min after bolus injection (G, H). Fluorescence lifetime images (blue-green-red) were recorded at λex/λem: 920/350 to 450 nm and λex/λem: 920/450 to 515 nm. Magnification: 40×. Scale bar: 40 μm.

Since EV-mediated chemotaxis has been associated with the establishment of the pre-metastatic niche in other cancers [39, 42], we investigated whether extracellular vesicles from highly metastatic OS clones could preferentially colonize the lungs, the most common site of distant metastasis in OS. To do so we used multiphoton microscopy with fluorescence lifetime imaging (MPM-FLIM) to track the distribution of PKH67-labelled HiMet-C6 extracellular vesicles in mice after intravenous injection. The fluorescence signals of PKH67-labelled extracellular vesicles in PBS *in vitro* could only be detected at 920 nm excitation and emission channel from 450 to 515 nm, with a fluorescence lifetime of 1922 ± 122 ns (Figure S4A and S4B). The pseudo-color was based on the average fluorescence lifetimes (τ_m_) in individual pixels. Figure 8 displays the spatial distribution of the fluorescence lifetime signal of PKH67-labelled extracellular vesicles in the mouse lung, as measured by MPM-FLIM at an excitation wavelength of 920 nm. In the 350–450 nm spectral channel, the fluorescence signal mainly comes from collagen second harmonic generation in the lung. As no fluorescence signal of PKH67-labelled extracellular vesicles was detected in this channel, the color and the average fluorescence lifetime (τ_m_) of images did not change significantly before (106 ± 14 ns) or after injection (108 ± 25 ns) as shown in Figure 8A and 8C. In sharp contrast, the spectral channel of 450–515 nm captured the fluorescence signals of PKH67-labelled extracellular vesicles as well as autofluorescence signals from flavin adenine dinucleotide in cells. The image color changed to orange in the pulmonary capillary (Figures 8B and 8D) after injection of PKH67-labelled extracellular vesicles and τ_m_ increased significantly (from 1089 ± 75 ns to 1663 ± 168 ns, *P* < 0.05) compared to the pre-injection value, demonstrating the localization of the fluorescence signals of PKH67-labelled extracellular vesicles in the lung. However, unlike the lung, the color and τ_m_ of images of liver did not change significantly before (Figure 8E and 8F) or after (Figures 8G and 8H) injection in all channels (728 ± 81 ns *vs.* 699 ± 49 ns, *P* > 0.05), which indicated that the fluorescence signals of PKH67-labelled extracellular vesicles in the liver were undetectable. Therefore, our *in vivo* data show the specific localization of HiMet-C6 extracellular vesicles to lung tissue which is in agreement with the clinical data showing the lung as the major site for the development of secondary metastases in OS.

## DISCUSSION

In this manuscript we show that EVs from highly metastatic OS clones are internalized by poorly metastatic clones and induce a migratory and invasive phenotype in the latter. Notably, this horizontal phenotypic transfer is unidirectional and provides definitive evidence that metastatic potential may arise *via* interclonal cooperativity mediated *via* secretion of EVs. In addition, we also show that the EVs derived from highly metastatic clones can release chemotactic factors that induce migration and invasion of poorly metastatic clones. This latter activity is of considerable interest since we also show that EVs from highly metastatic clones selectively concentrate within lung tissue where they may set up a chemotactic gradient to recruit OS cells to the pre-metastatic niche within the lung. Finally, we complete the first proteomic evaluation of the contents of secreted osteosarcoma EVs. We show that the amount and protein composition of EVs differs between highly- and poorly-metastatic clonal variants. In addition, we show that vesicles secreted by highly metastatic OS clonal variants carry a cargo of known metastasis drivers that operate through the regulation of G-protein coupled receptor (GPCR) signaling.

Although a number of studies have provided evidence for the EV-mediated transfer of oncogenic and metastatic properties between cancer cells that result in phenotypic changes in the recipient cells [33, 43–45], the literature has been lacking for osteosarcoma. In this study, we demonstrate that EVs, secreted by highly metastatic clonal subpopulations of OS, can be internalized by poorly metastatic subpopulations of OS. Moreover, this transfer of EVs results in a phenotypic change in the poorly metastatic subclones, making them significantly (*P* < 0.0001) more migratory and invasive. Importantly, this horizontal phenotypic transfer was only observed from highly metastatic subclones to poorly metastatic subclones, suggesting that a heterogeneous tumor could use this unidirectional EV-mediated interclonal cooperation specifically to promote its metastatic spread.

Our study is consistent with recent studies showing that interclonal cooperation can induce metastatic behavior in other tumour types [46, 47]. For example, it was recently revealed that EVs released by tumor cells can induce metastasis through the transfer of oncogenic and metastatic proteins and nucleic acids [33, 43–45]. For example, a truncated, oncogenic form of EGFR, EGFRvIII, can be transferred between glioma cells *via* EVs. This results in the transmission of oncogenic activity, including activation of MAPK and Akt signaling pathways, changes in expression of EGFRvIII-regulated genes, morphological transformation and an increase in anchorage-independent growth [33]. Finally, in breast cancer, EVs from invasive triple-negative breast cancer cells (TNBC) increase the invasiveness of less invasive TNBCs and of other non-TNBC cells [44].

The existence of circulating exosomes has been shown to increase with tumour progression and correlates with poor prognosis [20, 48–53]. Our data confirm that in OS, established metastatic cell lines and highly metastatic subpopulations secrete 3-fold more EVs than their non-metastatic or poorly metastatic counterparts. This suggests that levels of circulating EVs in OS patients could serve as a prognostic marker for metastatic progression. In this study, we reveal that in OS, EVs from highly metastatic tumor cell subpopulations carry a unique cargo of proteins not present in EVs from poorly metastatic subpopulations. Moreover, 16/31 (51.6%) of the unique highly metastatic EV proteins identified in our proteomic analysis have been shown to be associated with poor prognosis and/or metastatic progression (Table 1). Thus, our data suggests that some of these proteins could also serve as prognostic markers for metastatic OS. These include nucleophosmin (NPM1), vimentin (VIM) and valosin-containing protein (VCP). NPM1 has been directly implicated in tumor progression in a number of cancers [54]. VIM has been shown to increase cell motility, invasion and lamellipodia formation [55–57], and its expression is linked to increased metastatic progression, reduced patient survival and poor prognosis across multiple tumor types [58]. VCP also correlates with increasing recurrence and poor prognosis in patients with various cancers [59–65], and very recent studies have shown that its inhibition in osteosarcoma results in loss of malignancy and decreased metastasis [66–68]. Further investigation is needed to confirm if the other EV proteins identified in this study could also be relevant therapeutic targets for OS metastasis. As well as carrying proteins that directly impact metastatic progression, EVs have been shown to release chemokines that act as chemo-attractants and increase the chemotactic invasion of target cells, a process which can contribute to pre-metastatic niche formation [39, 69]. Consistent with this, we show that EVs from highly metastatic OS cell subpopulations have the capacity to increase chemotaxis and invasion of poorly metastatic cell subpopulations. This lends support to the proposition that interclonal cooperation is unidirectional, favoring the overall metastatic progression of a tumor, and possibly contributing to the establishment of a pre-metastatic niche. In support of the latter, we provide *in vivo* evidence to show that highly metastatic OS-derived EVs are selectively retained within the lung, the major and most common site of distant metastasis in OS, in preference to the liver, which is not a target organ for OS distant spread. Our observations are reinforced by very recent studies showing that tumor-derived EVs can seed and precondition specific organs for metastatic invasion [16, 70]. These studies indicate that vesicles derived from different cancer cells express different surface integrins which determine the homing of the vesicles to distant organs with an abundance of ligand for the particular integrin [16]. In addition, seeding of target organs by these EVs induces the production of S100 proteins which promote inflammation and cell migration [16]. Taken together, these findings may contribute to an explanation for lung tropism in OS.

## MATERIALS AND METHODS

### Cell culture

Osteosarcoma cells, KHOS (ATCC^®^ CRL-1544^™^), were a kind gift from Prof. Andreas Evdokiou, Basil Hetzel Institute, University of Adelaide, Australia. Cells were cultured in DMEM/10% FCS at 37°C/5%CO_2._ All cell lines were authenticated by Cell Bank Australia using PCR to identify short tandem repeats and match them to known ATCC profiles.

### Derivation of OS clonal variants and *in vivo* metastasis studies

The highly metastatic subclones, C1 and C6, and the poorly metastatic subclones, C4 and C5, were derived from KHOS as described. Briefly, cells were seeded in 24-well plates at a density of less than 1 cell/well. Individual cells were allowed to divide and grow into colonies over several days. Wells that contained growing colonies were expanded into tissue culture flasks and frozen in liquid nitrogen. Subclones were selected at random for injection into mice. KHOS cells were injected as a control. All animal experimentation was approved by, and carried out in strict accordance with the recommendations of, The University of Queensland Health Sciences Ethics Committee (Approval Numbers: UQDI/PAH/292/12/NHMRC). Four to six female 6-week-old BALB/c nude mice were used in each group and experiments were repeated at least once. Each subclone and KHOS was injected intra-femorally at a concentration of 5 × 10^6^ cells/mL in a 10 μL volume (50,000 cells). Mice were euthanized when tumors reached the ethics-imposed limit of 10 mm in diameter. Lungs were collected after perfusion with 4% paraformaldehyde, fixed, sectioned and H&E stained. Metastasis was quantified by measuring % area covered by metastatic lesions/% total lung area for each lung, using Nikon NIS-Elements software (Nikon Corporation Instruments, Tokyo, Japan), at 10× magnification.

### Isolation of extracellular vesicles

Medium free of fetal calf serum extracellular vesicles (FCS-EV-free medium) was prepared by centrifugation of DMEM/10% FCS overnight at 100,000 × *g*, at 4°C. The supernatant was collected without disturbing the pellet and filtered through a 0.2 um filter [71]. OS cells were seeded at 3 × 10^5^ cells/mL in 10 mL of DMEM/10% FCS and incubated overnight. Medium was then removed and the cells were washed three times in PBS before the addition of 12 mL of FCS-EV-free medium. Cells were incubated for 24 h. The medium was collected and centrifuged at 4000 rpm for 15 min to remove cells and debris. The supernatant was collected and filtered gently through a 0.2 μm membrane to remove debris. EV precipitation was performed on the tissue culture medium with ExoQuick-TC™ Exosome Precipitation Solution (System Biosciences) according to the manufacturer's instructions. For EVs isolated by serial ultracentrifugation, the cell culture medium was collected and centrifuged at 2000 × *g* for 10 min. The supernatant was then centrifuged at 10,000 × *g* for 10 min, and then concentrated in an Amicon Ultra-15 10kDa centrifugal filter, until 1.5 mL of supernatant remained. EVs were then pelleted at 100,000 × *g* for 70 min. All steps were performed at 4°C. The final pellet was washed in chilled PBS and then resuspended in 100 μL PBS for functional assays, or 100 μL RIPA buffer (50 mM Tris-HCl, pH 7.4, 150 mM NaCl, 0.1% SDS, 0.5% sodium deoxycholate, 1% NP-40) for immunoblotting and proteomic analysis. Protein was determined by the method of Bradford [72] using a dye reagent concentrate (Bio-Rad, Sydney, Australia). Extracellular vesicles were stored at −80°C in 20 μg aliquots until ready for use.

### Particle size analysis

EVs were diluted in PBS and particle size analysis was performed in a Zetasizer 300HSa (Malvern Instruments, UK) according to the manufacturer's instructions and using the following settings: size mode, rapid acquisition, 3 × 10 runs, Refractive Index = 1.331, viscosity = 0.89, temperature = 25C.

### Electron microscopy

Aliquots of EVs in PBS and 8% EM grade paraformaldehyde/PBS were mixed in equal volumes and fixed in 4% EM-grade paraformaldehyde. The preparations were randomly adsorbed onto carbon-coated Formvar films on copper grids by floating a dry grid on a 5 μl drop of the microvesicle suspension for 15 min. Grids were washed 7 times with ultrapure water and stained with 0.4% uranyl acetate in 2% methylcellulose for 5 min on ice. Grids were dried and visualized at 60,000× in a transmission electron microscope (JEOL model 1011, USA) equipped with a Morada side-mounted camera with a Peltier-cooled CCD chip (Olympus, Germany), and iTEM software (Olympus).

### Immunoblotting

EV pellets were resuspended in RIPA buffer and protein assays were performed as above (BioRad). Samples were run on a 10% SDS-PAGE gel. Proteins were detected with rabbit antibody to CD63, CD9, CD81 and HSP70 (EXOAB-KIT-1, System Biosciences, CA, USA), 1:1000.

### Extracellular vesicle labelling and uptake assay

EV membranes were labelled with PKH67 dye (Sigma) following the manufacturer's instructions. Briefly, 20 μg of EVs were labelled with dye for 5 min and the reaction stopped with 1% BSA. EVs were centrifuged at 100,000 × *g* for 2 h at 4°C. The supernatant was removed and the pellet was washed three times in 1% BSA to remove unbound dye. The centrifugation and washing steps were repeated and the final pellet was washed in BSA and PBS, and resuspended in 100 μL PBS before protein quantification. Cells were seeded at 1 × 10^5^cells/mL onto coverslips in a 6-well plate and incubated at 37°C overnight until 60% confluent. The medium was replaced with FCS EV-free medium containing 20 μg/mL of PKH67-labelled OS EVs. Cells were incubated for 2, 4 and 20 h. Cells were washed 3 times with PBS to remove unattached vesicles and fixed with 4% paraformaldehyde. Coverslips were washed again and stained with 0.1 μg/mL DAPI (Cell Signaling Technology, USA) before mounting. Visualization was performed by confocal microscopy at 63× magnification (Zeiss LSM 510 Meta, Germany).

### Migration, invasion and chemotaxis assays

Assays were performed in 24 mm Transwell plates with 8.0 μm pore polycarbonate membrane inserts (Corning Life Sciences, USA). For migration assays the membranes were uncoated. For invasion assays the membranes were coated with 50 μL of Matrigel^TM^ (BD Biosciences, USA), diluted 1:4 and solidified by incubation at 37°C for at least 30 min. OS cells were seeded in serum-free DMEM into the upper chamber of inserts at a concentration of 2 × 10^5^ cells/mL for migration assays or 1 × 10^6^ cells/mL for invasion assays, in the presence or absence of extracellular vesicles (0, 20 and 40 μg/mL). Cells were allowed to migrate for 24 h (migration) or 48 h (invasion) towards DMEM/20% FCS in the lower chamber. For chemotaxis assays [73, 74], the extracellular vesicles were placed in the bottom chamber at a concentration of 20 μg/mL. Migrated cells were labelled with 8 μM calcein-AM (Sigma-Aldrich) and detached with trypsin-EDTA. Fluorescence was measured in a FLUOstar Optima (BMG Labtech, Germany) at 485 nm (excitation) and 520 nm (emission).

### Proliferation assay

Cells were diluted to 5 × 10^4^ cells/mL and divided into 500 uL aliquots. The aliquots were centrifuged and the medium was replaced with 500 uL DMEM/10% FCS containing 0, 20 or 40 μg/mL of EVs. Samples (100 μL/well) were added into a 96-well plate in triplicate. Plates were incubated at 37°C for 24 hours. Cell proliferation was quantified using a commercial colorimetric immunoassay based on the measurement of BrdU incorporation during DNA synthesis (Roche Diagnostics, Germany), following the manufacturer's protocol.

### Proteomic analysis

EVs were isolated from filtered HiMet-C6 and LoMet-C4 cell culture medium as described above. The EV pellet was resuspended in RIPA buffer and protein concentration was determined as above. The EV protein was mixed with 4× protein loading buffer (200 mM Tris-HCl, pH 6.8, 8% SDS, 40% glycerol, 400 mM DTT, 0.4% bromophenol blue) and stored at −20°C until ready for analysis. Three biological replicates containing equal amounts of protein were run for each EV preparation. The protein concentration of HiMet-C6 and LoMet-C4 samples used for proteomic analysis were the same. Samples were separated by SDS PAGE to 8 mm. The gels were stained overnight with colloidal Coomassie and destained with 1% acetic acid. The protein samples were divided into 8 × 1 mm gel bands. The protein gel bands were subjected to robot assisted in-gel digest (Bravo Automated Liquid Handling Platform, Agilent). The extracted peptides were dried and resuspended in 5% formic acid for mass spectrometry (MS) analysis. Each peptide fraction was loaded onto a G4240-62010 large capacity chip (Agilent) and analyzed by MS (HPLC-Chip/MS with 6520Accurate Mass Q-TOF LC/MS, Agilent). Data was processed with Spectrum Mill Rev B.04.00.127 (Agilent). Data were extracted using standard settings and searched against Swiss Prot Human (version 11/2014). Search parameters were set to digest with trypsin with a maximum of 2 miscleavages. Peptide modifications were set to fix carbamidomethylation C and variable oxidized M. Tolerance was set to 50% minimum matched peak intensity, with 20 ppm and 50 ppm precursor and product mass tolerance respectively. Proteins were summarized from IDs with the following cut-off settings: protein score of > 11, peptide score> 10, 60% scored peak intensity and false discovery rate (FDR) of > 0.5%.

### Multiphoton microscopy with fluorescence lifetime imaging

Multiphoton microscopy (MPM) was performed using the DermaInspect system (Jen-Lab GmbH, Jena, Germany) equipped with an ultrashort (85 fs pulse width) pulsed mode-locked 80-MHz titanium sapphire laser (MaiTai, Spectra Physics, Santa Clara, CA, USA). For fluorescence lifetime imaging (FLIM), a time-correlated single-photon counting (TCSPC) SPC-830 detector (Becker & Hickl, Berlin, Germany) was incorporated into the multiphoton microscopy system. The TCSPC module constructs a photon distribution across the x and y coordinates of the scan area. In MPM-FLIM images, the fluorescence decay kinetics of each individual pixel are modelled to yield a spatial distribution of fluorescence lifetime. This enables the differentiation of fluorophores and their environments in biological tissues. Fluorescence emission was spectrally resolved between linearly arranged photon counters through the use of dichroic filters in the beam path. The excitation wavelength was set to 920 nm for signals of PKH67-labelled extracellular vesicles, with an emission signal range of 450 to 515 nm established through the use of a BG39 bandpass filter (BG39, Schott glass color filter, Schott MG, Mainz, Germany). The emission light at 920-nm excitation was collected spectrally in a channel from 350 to 450 nm for second harmonic generation (SHG). Images were recorded with oil-immersion 40× objectives (Carl Zeiss, Germany). The laser power was set to 15 mW for imaging the lung, and 35 mW for the liver, and each FLIM scan was performed using an exposure of 13.4 s.

Mice were anaesthetized initially by an intraperitoneal injection of ketamine hydrochloride (80 mg/kg) and xylazine (10 mg/kg). Body temperature was controlled by placing mice on a heating pad set to 37°C. A thoracotomy or laparotomy was performed to expose the lung or liver. MPM-FLIM images of unfixed live lungs or livers were collected within 30 min after surgical procedures started. Normal saline was used to keep the lung or liver moist and attached to the cover glass of ring interfaced to multiphoton microscopy throughout the experiment. Twelve images from twelve non-overlapping fields were collected per mouse.

### Analysis of imaging data

FLIM images were analyzed using SPCImage software 4.9.7 (Becker & Hickl, Berlin, Germany). A bin of two was used in all images when smoothing the decay data prior to fitting. The decay curve is a sum of multiple components as each pixel represents an overlay of emissions from various fluorophores. In this study, a bi-exponential decay model function was used where τm represents the weighted average lifetime of the acquisition image.

### Statistical analysis

Statistical tests were performed in GraphPad Prism by either one-way ANOVA, with Tukey's Multiple Comparison Test for a significance level of *P* < 0.05, or column analysis and parametric, un-paired, two-tailed *t*-test analysis.

## SUPPLEMENTARY MATERIAL FIGURES AND TABLES


