# Supplementary material for: Extracellular vesicles secreted by highly metastatic clonal variants of osteosarcoma preferentially localize to the lungs and induce metastatic behaviour in poorly metastatic clones
Source: Oncotarget. 2016 Jun 2;7(28):43570–87. doi: 10.18632/oncotarget.9781 (PMC5190045; doi:10.18632/oncotarget.9781)
Supplement: Supplementary file 1 [file oncotarget-07-43570-s001.pdf]

Extracellular vesicles secreted by highly metastatic clonal variants of osteosarcoma preferentially localize to the lungs and induce metastatic behaviour in poorly metastatic clones

Supplementary Materials

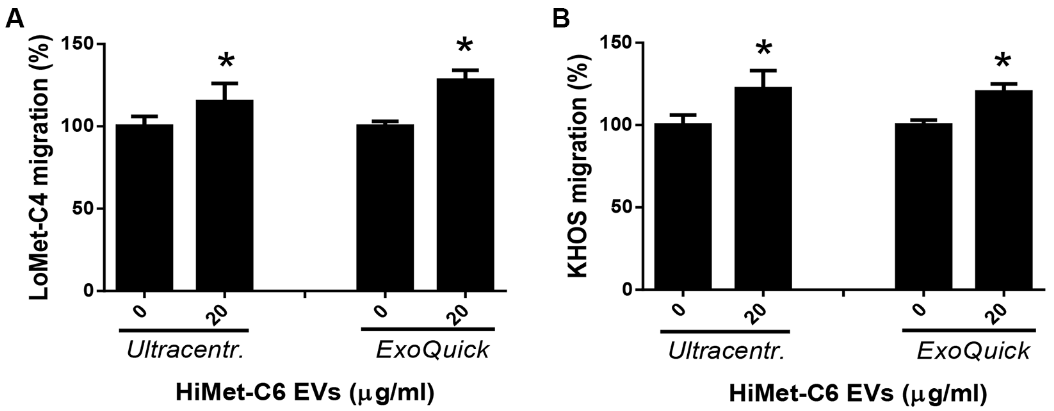

Supplementary Figure S1: Extracellular vesicles purified by ultracentifugation or ExoQuick are functionally similar. EVs were isolated from serum free and EV-free media derived from HiMet-C6 clones by ultracentrifugation (Ultracentr.) or ExoQick TC Exosome Precipitation Solution (System Biosciences) (ExoQuick). Migration of LoMet-C4 clones (**A**) or parental KHOS cells (**B**) was estimated in the presence of no EVs (0) or 20 µg / ml EVs (20). Data presented as mean +/− SEM from triplicate determinations of 3 independent experiments. *$P < 0.05$ from ANOVA.

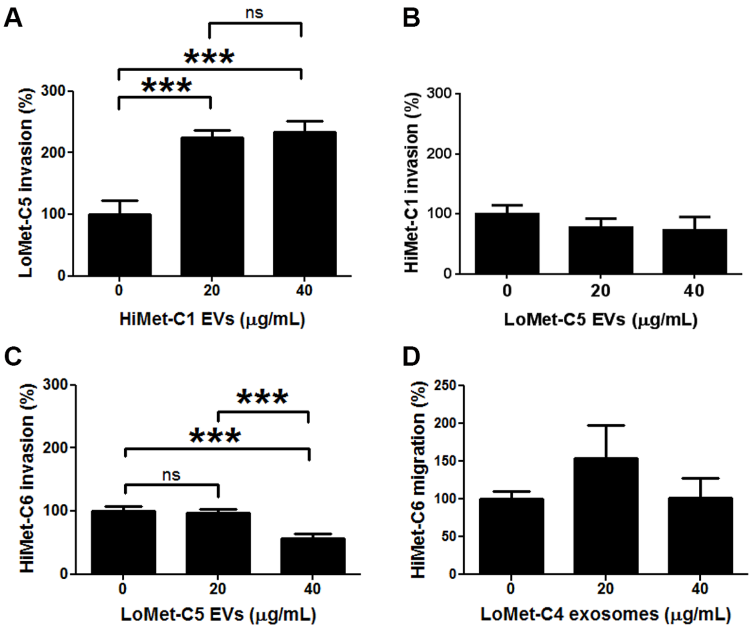

Supplementary Figure S2: Extracellular vesicles secreted by LoMet clonal variants do not increase migration or invasion of HiMet clonal variants. (**A**) Increase in the invasion of LoMet clonal variant C5 in the presence of EVs secreted by HiMet clonal variant C1. *1-way ANOVA (F = 102, $P < 0.0001$), Tukey's MCT for $P < 0.05$. (**B**, **C**, **D**) EVs secreted by LoMet EVs (C4, C5) do not increase migration or invasion of HiMet clonal variants (C1, C6). Statistical analysis: (B) 1-way ANOVA (F = 3.58, $P = 0.055$), Tukey's MCT for $P < 0.05$ = ns; (C) 1-way ANOVA (F = 15.8, $P = 0.0003$), Tukey's MCT for $P < 0.05$. (D) 1-way ANOVA (F = 0.99, $P = 0.397$), Tukey's MCT for $P < 0.05$ = ns. Experiments were performed in triplicate at least twice. Bars: SD.

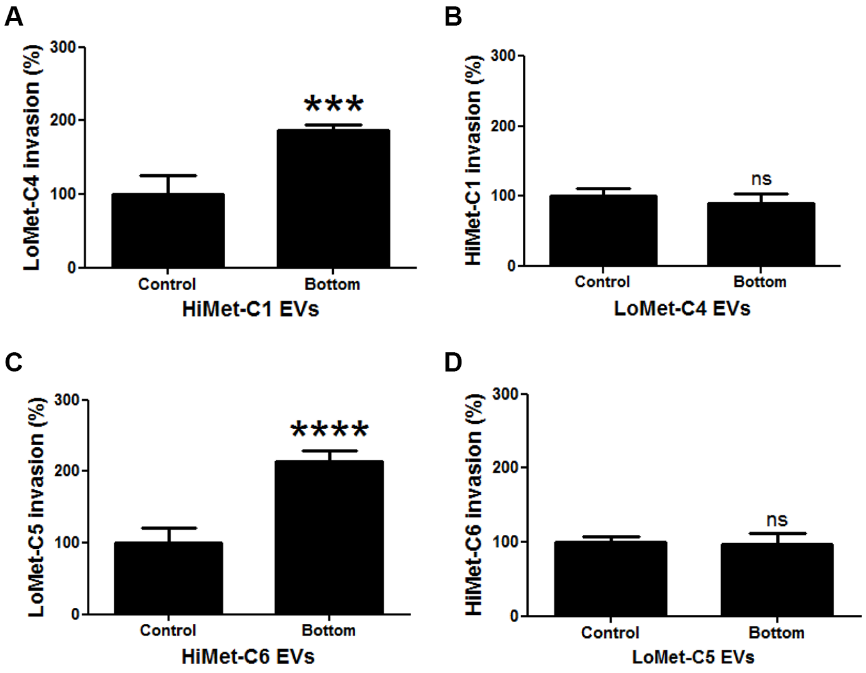

Supplementary Figure S3: Chemotactic properties of HiMet EVs. (**A**) Increase in the migration of LoMet-C4 in response to HiMet-C1 EV chemotaxis. ***$P = 0.0003$. (**B**) LoMet-C4 EVs do not induce an increase in the migration of HiMet-C1 cells by chemotaxis. (**C**) Increase in the invasion of LoMet-C5 in response to HiMet-C6 EV chemotaxis. ****$P < 0.0001$. (**D**) LoMet-C5 EVs do not induce a response in invasion by HiMet-C6 cells by chemotaxis. Experiments were performed in triplicate at least twice. Two-tailed *P* value from an unpaired *t* test. Bars: SD.

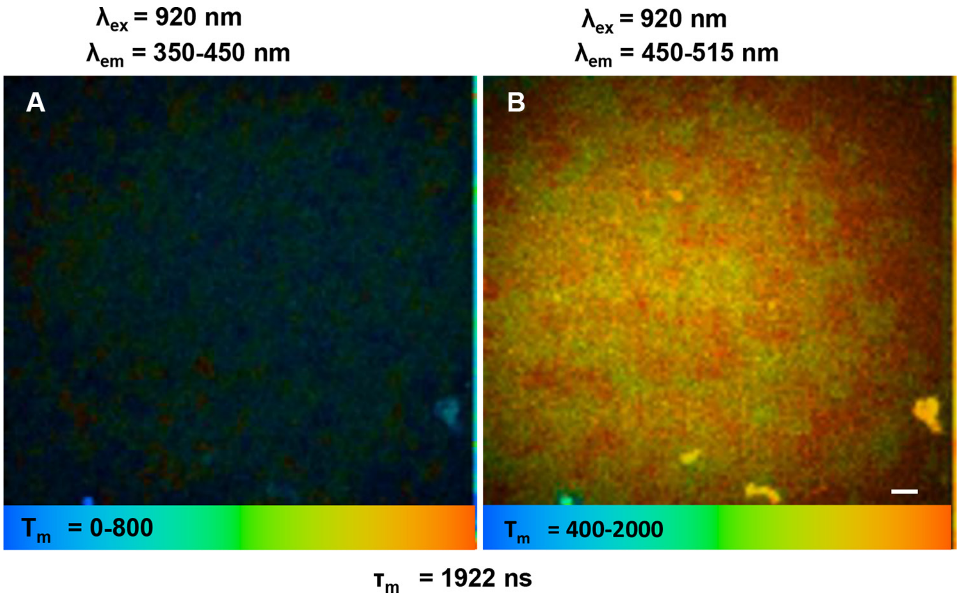

Supplementary Figure S4: MPM-FLIM images of PKH67-labelled HiMet-C6 EVs in PBS. (**A**) Pseudocolored τ_m fluorescence lifetime image (0–800 ps; blue-green-red) recorded at $\lambda_{Exc}/\lambda_{Em}$: 920/350 to 450 nm. (**B**) Pseudocolored τm fluorescence lifetime image (400–2000 ps; blue-green-red) recorded at $\lambda_{Exc}/\lambda_{Em}$: 920/450 to 515 nm. Magnification: 40×. Scale bar: 40 µm.

Supplementary Table S1: Proteomic analysis of HiMet-C6 EVs

Accession No.	Symbol UniProtKB	Entry Name	Ave. Total Intensity
P62263	RPS14	40S ribosomal protein S14	1.60E + 05
P62249	RPS16	40S ribosomal protein S16	1.20E + 06
P15880	RPS2	40S ribosomal protein S2	4.52E + 05
P62701	RPS4X	40S ribosomal protein S4, X isoform	2.46E + 05
Q8NHW5|P05388	RPLP0P6	60S acidic ribosomal protein P0-like	2.28E + 05
P50914	RPL14	60S ribosomal protein L14	5.72E + 04
Q02878	RPL6	60S ribosomal protein L6	1.82E + 05
P63261	ACTG1	Actin, cytoplasmic 2	2.25E + 07
P01023|P20742	A2M	Alpha-2-macroglobulin	1.52E + 08
O43707|P12814	ACTN4	Alpha-actinin-4	4.35E + 05
P02771	AFP	Alpha-fetoprotein	3.01E + 06
P04083	ANXA1	Annexin A1	1.55E + 05
P07355|A6NMY6	ANXA2	Annexin A2	9.25E + 05
P02647	APOA1	Apolipoprotein A-I	9.60E + 05
P04114	APOB	Apolipoprotein B-100	2.91E + 07
P53396	ACLY	ATP-citrate synthase	8.33E + 04
Q00610	CLTC	Clathrin heavy chain 1	7.23E + 05
P12259	F5	Coagulation factor V	3.68E + 04
P12109	COL6A1	Collagen alpha-1(VI) chain	2.71E + 06
P12110	COL6A2	Collagen alpha-2(VI) chain	6.53E + 05
Q9BXJ4	C1QTNF3	Complement C1q tumor necrosis factor-related protein 3	9.07E + 05
P01024	C3	Complement C3	3.18E + 07
P0C0L4|P0C0L5	C4A	Complement C4-A	2.20E + 07
Q9Y240	CLEC11A	C-type lectin domain family 11 member A	1.87E + 05
Q5VTE0|P68104	EEF1A1P5	Elongation factor 1-alpha 1	4.77E + 06
O00303	EIF3F	Eukaryotic translation initiation factor 3 subunit F	1.39E + 05
P49327	FASN	Fatty acid synthase	4.76E + 05
P02751	FN1	Fibronectin	4.01E + 06
P23142	FBLN1	Fibulin-1	4.36E + 06
P06396	GSN	Gelsolin	2.92E + 05
P04406	GAPDH	Glyceraldehyde-3-phosphate dehydrogenase	3.47E + 06
P07900|P08238	HSP90	Heat shock protein HSP 90-alpha	3.50E + 06
P69905	HBA1	Hemoglobin subunit alpha	1.99E + 07
P02100|P69891	HBE1	Hemoglobin subunit epsilon	2.14E + 07
P07910|B7ZW38	HNRNPC	Heterogeneous nuclear ribonucleoproteins C1/C2	5.06E + 05
P04908|Q7L7L0	HIST1H2AB	Histone H2A type 1-B/E	4.63E + 06
Q99879|Q99880	HIST1H2BM	Histone H2B type 1-M	2.12E + 06
Q71DI3	HIST2H3A	Histone H3.2	2.70E + 06
P62805	HIST1H4A	Histone H4	6.43E + 06
Q06033	ITIH3	Inter-alpha-trypsin inhibitor heavy chain H3	1.71E + 06
P04264|P35908	KRT1	Keratin, type II cytoskeletal 1	1.05E + 06
P51884	LUM	Lumican	6.50E + 05
P22234	PAICS	Multifunctional protein ADE2	3.05E + 05
P35580|P35579	MYH10	Myosin-10	4.17E + 05
P06748	NPM1	Nucleophosmin	1.63E + 06

P36955	PEDF/SERPINF1	Pigment epithelium-derived factor	1.43E + 06
P00747	PLG	Plasminogen	4.33E + 06
P60900	PSMA6	Proteasome subunit alpha type-6	1.03E + 06
P0DME0|Q01105	SETSIP	Protein SETSIP	3.28E + 05
P00734	F2	Prothrombin	1.49E + 06
P14618	PKM	Pyruvate kinase PKM	4.38E + 05
P02768	ALB	Serum albumin	1.91E + 08
P62314	SNRPD1	Small nuclear ribonucleoprotein Sm D1	1.88E + 05
P78371	CCT2	T-complex protein 1 subunit beta	1.42E + 06
P50991	CCT4	T-complex protein 1 subunit delta	7.71E + 05
P50990	CCT8	T-complex protein 1 subunit theta	1.40E + 06
P40227	CCT6A	T-complex protein 1 subunit zeta	2.61E + 05
P07996	THBS1	Thrombospondin-1	8.23E + 06
P35443|P49747	THBS4	Thrombospondin-4	4.08E + 05
P05543	SERPINA7	Thyroxine-binding globulin	5.76E + 05
P55072	VCP	Valosin containing protein	2.76E + 05
P68363|Q9NY65	TUBA1B	Tubulin alpha-1B chain	3.06E + 06
P08670	VIM	Vimentin	8.65E + 05
P04004	VTN	Vitronectin	6.68E + 05

Proteins expressed in at least 2 of 3 biological replicates analysed in 3 independent runs.

Supplementary Table S2: Proteomic analysis of LoMet-C4 EVs

Accession No.	Symbol UniProtKB	Entry Name	Ave. Total Intensity
Q15008	PSMD6	26S proteasome non-ATPase regulatory subunit 6	3.00E + 05
P61313	RPL15	60S ribosomal protein L15	7.21E + 04
P63261|P60709	ACTG1	Actin, cytoplasmic 2	1.95E + 07
P01023|P20742	A2M	Alpha-2-macroglobulin	7.86E + 07
P02771	AFP	Alpha-fetoprotein	1.09E + 06
P07355|A6NMY6	ANXA2	Annexin A2	1.49E + 06
P02647	APOA1	Apolipoprotein A-I	1.14E + 06
P04114	APOB	Apolipoprotein B-100	4.15E + 07
P12109	COL6A1	Collagen alpha-1(VI) chain	3.83E + 06
P12111	COL6A3	Collagen alpha-3(VI) chain	1.74E + 05
Q9BXJ4	C1QTNF3	Complement C1q tumor necrosis factor-related protein 3	8.97E + 05
P01024	C3	Complement C3	2.10E + 07
P0C0L4|P0C0L5	C4A	Complement C4-A	2.68E + 07
P08603	CFH	Complement factor H	2.25E + 06
Q5VTE0|P68104	EEF1A1P5	Elongation factor 1-alpha 1	2.85E + 06
P49327	FASN	Fatty acid synthase	6.68E + 05
P02751	FN1	Fibronectin	4.08E + 06
P23142	FBLN1	Fibulin-1	5.85E + 06
P06396	GSN	Gelsolin	2.60E + 05
P04406	GAPDH	Glyceraldehyde-3-phosphate dehydrogenase	1.31E + 06
P07900|P08238	HSP90	Heat shock protein HSP 90-alpha	2.81E + 06
P69905	HBA1	Hemoglobin subunit alpha	9.54E + 06
P02100|P69891	HBE1	Hemoglobin subunit epsilon	1.58E + 07
P04908|Q7L7L0	HIST1H2AB	Histone H2A type 1-B/E	1.30E + 06

P62805	HIST1H4A	Histone H4	5.77E + 05
Q06033	ITIH3	Inter-alpha-trypsin inhibitor heavy chain H3	4.71E + 05
P04264	KRT1	Keratin, type II cytoskeletal 1	7.16E + 05
P51884	LUM	Lumican	3.38E + 05
P36955	PEDF/SERPINF1	Pigment epithelium-derived factor	2.70E + 05
P00747	PLG	Plasminogen	6.20E + 06
P60900	PSMA6	Proteasome subunit alpha type-6	5.23E + 05
P00734	F2	Prothrombin	9.00E + 05
P02768	ALB	Serum albumin	1.56E + 08
P07996	THBS1	Thrombospondin-1	4.23E + 06
P35443|P49747	THBS4	Thrombospondin-4	4.64E + 05
P68363|P68366	TUBA1B	Tubulin alpha-1B chain	3.26E + 06
P04004	VTN	Vitronectin	9.95E + 05

Proteins abundant in at least 2 of 3 biological replicates analysed in three independent runs.

Supplementary Table S3: Proteins in HiMet-C6 EVs grouped according to biological process

GO – Biological process	UniProtKB	Symbol	Name	Subcellular localization
Vesicle trafficking, fusion, exocytosis, endocytosis, cellular transport	O43707	ACTN4	Alpha-actinin 4	Nucleus, cytoplasm
	P04083	ANXA1	Annexin A1	Nucleus, cytoplasm
	P78371	CCT2	T-complex protein 1 subunit beta	Cytoplasm
	P50991	CCT4	T-complex protein 1 subunit delta	Cytoplasm
	P40227	CCT6A	T-complex protein 1 subunit zeta	Cytoplasm
	P50990	CCT8	T-complex protein 1 subunit theta	Cytoplasm
	Q00610	CLTC	Clathrin heavy chain 1	Cytoplasm
	P69905	HBA1	Hemoglobin subunit alpha	Cytosol
	P55072	VCP	Vasolin containing protein	Cytoplasm, ER
	P05543	SERPINA7	Thyroxine-binding globulin	Secreted, extracellular exosome
Transcription regulation, DNA repair, DNA replication, chromosomal stability, nucleosome remodeling	O00303	EIF3F	Eukaryotic translation initiation factor 3 subunit F	Cytoplasm
	P04908	HIST1H2AB	Histone H2A type 1-B/E	Nucleus
	Q99879	HIST1H2BM	Histone H2B type 1-M	Nucleus
	P62805	HIST1H4A	Histone H4	Nucleus
	Q71DI3	HIST2H3A	Histone H3.2	Nucleus
	P07910	HNRNPC	Heterogeneous nuclear ribonucleoproteins C1/C2	Nucleus
	P06748	NPM1	Nucleophosmin	Nucleus, cytoplasm
	P0DME0	SETSIP	Protein SETSIP	Cytoplasm, nucleus
RNA binding, translation	P50914	RPL14	60S ribosomal protein L14	Cytoplasm, membrane
	Q02878	RPL6	60S ribosomal protein L6	Cytosol, membrane, nucleus
	Q8NHW5	RPLP0P6	60S acidic ribosomal protein P0-like	Cytosol
	P62263	RPS14	40S ribosomal protein S14	Cytosol, cytoplasm, membrane, mitochondria
	P62249	RPS16	40S ribosomal protein S16	Cytosol, membrane
	P15880	RPS2	40S ribosomal protein S2	Cytoplasm, membrane, nucleus
	P62701	RPS4X	40S ribosomal protein S4, X isoform	Cytoplasm
	P62314	SNRPD1	Small nuclear ribonucleoprotein Sm D1	

Cytoskeleton remodeling, ECM proteolysis, cell migration	O43707	ACTN4	Alpha-actinin 4	Nucleus, cytoplasm
	P12110	COL6A2	Collagen alpha-2(VI) chain	Membrane, secreted
	P12259	F5	Coagulation factor V	Extracellular region
	P04406	GAPDH	Glyceraldehyde-3-phosphate dehydrogenase	Cytoplasm, nucleus, membrane
	Q06033	ITIH3	Inter-alpha-trypsin inhibitor heavy chain H3	Extracellular region
	P35580	MYH10	Myosin-10	Extracellular region
	P36955	PEDF/ SERPINF1	Pigment epithelium-derived factor	Extracellular region
	P08670	VIM	Vimentin	Cytoplasm
Metabolism, ion transport, tumour cell proliferation and survival	P53396	ACLY	ATP-citrate synthase	Cytoplasm
	P02771	AFP	Alpha-fetoprotein	Cytoplasm, extracellular space
	P22234	PAICS	Multifunctional protein ADE2	Cytosol, membrane
	P14618	PKM	Pyruvate kinase PKM	Cytoplasm, nucleus

To identify function (as GO-Biological Process), the Universal Protein Resource (UniProt) database was interrogated using the accession number for each of the 38 peptides identified in the analysis as abundant in HiMet EVs (FDR = 0.5%).
